# Single-molecule analysis reveals that a glucagon-bound extracellular domain of the glucagon receptor is dynamic

**DOI:** 10.1016/j.jbc.2023.105160

**Published:** 2023-08-14

**Authors:** Ting Liu, Susmita Khanal, Gillian D. Hertslet, Rajan Lamichhane

**Affiliations:** Department of Biochemistry & Cellular and Molecular Biology, College of Arts & Sciences, University of Tennessee, Knoxville, Tennessee, USA

**Keywords:** G protein-coupled receptors, glucagon receptor, conformational dynamics, single-molecule, FRET, micelle, biophysics, membrane proteins

## Abstract

Dynamic information is vital to understanding the activation mechanism of G protein-coupled receptors (GPCRs). Despite the availability of high-resolution structures of different conformational states, the dynamics of those states at the molecular level are poorly understood. Here, we used total internal reflection fluorescence microscopy to study the extracellular domain (ECD) of the glucagon receptor (GCGR), a class B family GPCR that controls glucose homeostasis. Single-molecule fluorescence resonance energy transfer was used to observe the ECD dynamics of GCGR molecules expressed and purified from mammalian cells. We observed that for apo-GCGR, the ECD is dynamic and spent time predominantly in a closed conformation. In the presence of glucagon, the ECD is wide open and also shows more dynamic behavior than apo-GCGR, a finding that was not previously reported. These results suggest that both apo-GCGR and glucagon-bound GCGRs show reversible opening and closing of the ECD with respect to the seven-transmembrane (7TM) domain. This work demonstrates a molecular approach to visualizing the dynamics of the GCGR ECD and provides a foundation for understanding the conformational changes underlying GPCR activation, which is critical in the development of new therapeutics.

The glucagon receptor (GCGR) is a class B family G protein-coupled receptor (GPCR) activated by the 29 amino-acid peptide hormone glucagon, which is released from the α-cells of the islet of Langerhans. The activated GCGR promotes downstream signaling by interacting with the stimulatory G protein (G_s_) *via* adenylyl cyclase. The downstream signaling cascade mediates the release of glucose from the liver into the bloodstream. Because of its role in regulating blood glucose levels, GCGR is a therapeutic target for treating type 2 diabetes. Several studies in animal models have shown that type 1 diabetes was attenuated by treating the animal with GCGR antagonists or genetic deletion of the GCGR (1, 2). However, other studies have shown that fulminant diabetes will still develop in mice that lack insulin despite the absence of GCGR signaling (3, 4, 5). Interestingly, GCGR has also been reported to play an important role in regulating type 2, or noninsulin-dependent diabetes mellitus, by reducing its expression or activity through GCGR antagonist (6, 7, 8). Moreover, lack of suppression of glucagon leads to postprandial hyperglycemia (9, 10). Studies have shown that GCGR antagonists inhibits glucagon-mediated glycogenolysis (11, 12). *In vivo* studies have also shown that the reduction of GCGR expression benefits the treatment of diabetes (6, 13). In this regard, the development of a GCGR inhibitor is significant, especially in understanding how this inhibitor interacts with GCGR to inhibit the downstream signaling, eventually blocking the development of diabetes. The extracellular domain (ECD) has been shown to act an intrinsic negative regulator of GCGR activity; the mechanisms of GCGR inhibition were indicated by blocking antibodies (mAb1 and mAb23) targeting the ECD (14). In this regard, development of structural and functional GCGR antagonists represents a potential approach to decrease hepatic glucose production and lower blood glucose in patients with diabetes. Meanwhile, a deeper understanding of GCGR activity and its modulatory mechanisms are critical for developing new therapeutics.

The crystal and cryo-EM structures of GPCR complexes with drugs and associated proteins have successfully been determined in the past decades, which have greatly expanded our understanding of GPCR biology. The conformational dynamics of GPCRs have been investigated by multiple technologies like NMR spectroscopy, double electron-electron resonance spectroscopy, fluorescent-based assays, and computational approaches (15, 16, 17, 18), indicating that GPCRs are highly dynamic and populate multiple conformational states. Previous studies have suggested that peptide ligands binding to class B GPCRs follow a two-domain binding model, in which the C-terminal portion of the peptide targets the ECD and the N-terminal portion of the peptide binds to the transmembrane domain (TMD) binding pocket, leading to conformational rearrangements and receptor activation (19, 20, 21). However, a study showed that the ECD is required for receptor signaling even when the ligand is tethered to the TMD, complicating the “two-domain” model (22). Recent studies have focused on understanding the structural plasticity of glucagon receptor and mechanisms of signal transduction in complex with a glucagon analog. This suggests the conformational changes in the ECD, stalk region, and the first extracellular loop play an important role during peptide binding (23, 24, 25). A large number of structures for class B GPCRs are available showing the full-length receptors and interacting functional peptide hormones. These structures have clearly identified open and closed states of the ECD in their apo form and in complex with the ligands (24, 25, 26, 27). Moreover, computational studies have predicted that the ECD of class B GPCRs access both open and closed conformations even without ligand binding (23, 26). However, much less is known about the ECD dynamics and the transitions among different conformational states at the molecular level.

To elucidate the molecular details governing peptide binding and ECD dynamics, we use single-molecule fluorescence resonance energy transfer (smFRET), as it has become a leading method for determining dynamic information of biomolecular complexes *in vitro* and *in vivo* (28, 29, 30, 31, 32). smFRET has been used previously to study structural dynamics and activation of class A and class C GPCRs (33, 34, 35, 36), but it has never been used to study the ECD dynamics in class B GPCRs (35). In this study, we purified GCGR from mammalian cells and used smFRET to observe ECD dynamics in the presence and absence of GCGR ligands. Our data show that for apo-GCGR, the ECD transitions between at least two conformational states, which is consistent with available structures of GCGR and its homolog GLP-1R (glucagon-like peptide 1) alone or in complex with peptide ligands and antibodies (23, 24, 26, 37). However, in the presence of the agonist glucagon, the ECD moves away from the 7TM, making the peptide ligand binding pocket more open. Interestingly, our data reveal that the glucagon-bound GCGR ECD still fluctuates among multiple conformational states, which was not explored previously at the molecular level. This study provides a structural basis for further investigating the role of the ECD in ligand binding and receptor dynamics.

## Results

### Detergent purification and characterization of GCGR expressed in HEK293T cells

A C-terminal hexa-histidine-tagged (6xHis) and FLAG-tagged human GCGR was expressed in HEK293T cells and solubilized in a buffer containing 0.05% (w/v) n-dodecyl-beta-D-maltopyranoside (DDM) and 0.01% (w/v) cholesteryl hemisuccinate (CHS). Nonsolubilized material was removed by centrifugation, and GCGR–micelle was purified using a Ni^2+^-NTA resin. Purified protein was confirmed by Coomassie blue staining and Western blot using an anti-FLAG tagged antibody (Fig. S1*A*). To examine GCGR in the micelle, we performed mass photometry experiments and observed a mass distribution of around 64 kDa (Fig. S1*B*) for the majority (60%) of GCGR–micelle molecules. To test whether the purified and labeled GCGR in the micelle retains functional activity, we performed a fluorescence-based ligand-binding assay. For this, we expressed and site-specifically labeled GCGR at position 287 (Cys287) with a FRET donor (Alexa Fluor 555) and the glucagon peptide with FRET acceptor (Alexa Fluor 647) at Cys28 (Fig. S2). Control experiments were done to show that labels did not affect the ligand-binding activity (Supporting Text and Fig. S3).

### smFRET reveals the dynamic nature of GCGR ECD

**Figure 1:**
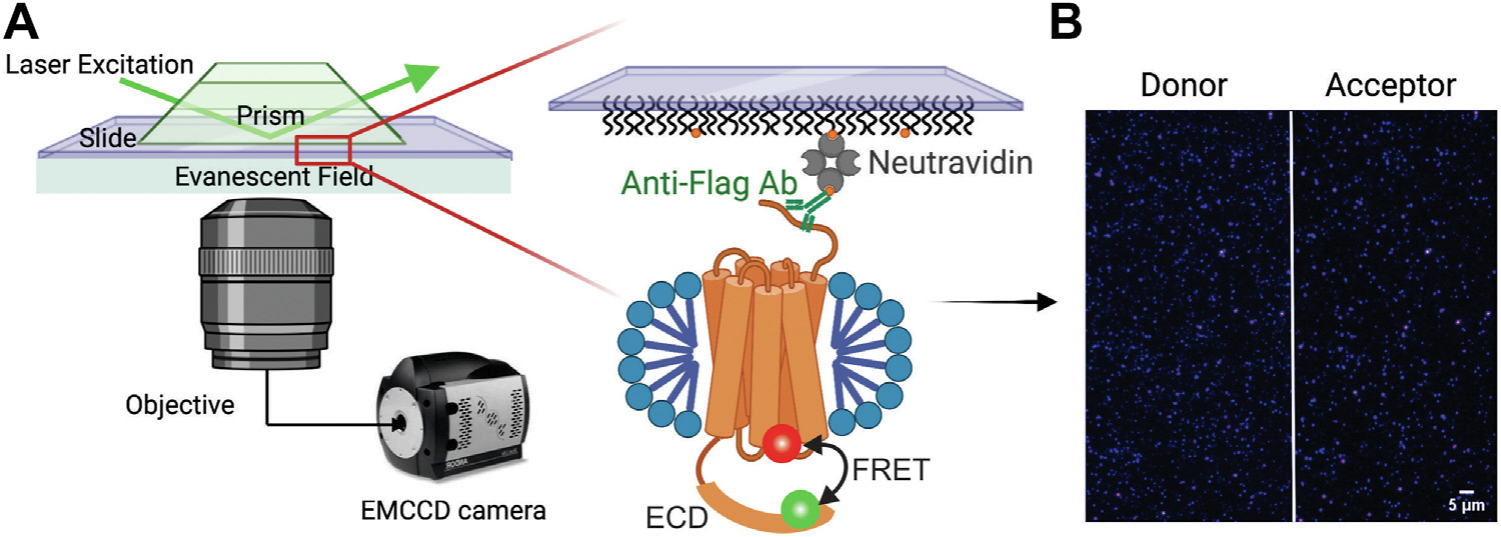
**Schematic diagram of prism-based single-molecular total internal reflection fluorescence (TIRF) microscopy and representative TIRF field-of-view image with the donor and acceptor channels.***A*, schematic of single-molecule TIRF imaging of GCGR in detergent micelle labeled with donor and acceptor fluorophores. The excitation beam reaches the slide-solution interface and creates an evanescent wave that excites immobilized molecules in an aqueous solution. The emission from the donor and acceptor are collected through an inverted microscope objective and passed through a dual-view splitter, where the donor (*green*) and acceptor (*red*) emissions are physically separated. Finally, the two emission signals are detected side-by-side by an electron-multiplied back-illuminated CCD camera. The expanded view showing Alexa Fluor 555- (*green sphere*) and Alexa Fluor 647- (*red sphere*) labeled GCGR (*orange cylinders*) in a detergent micelle (*blue*) immobilized to a microscope slide using biotin (*orange circles*), streptavidin (*gray semicircles*) and biotinylated anti-FLAG antibody (*green Y shape*). The microscope slides were passivated with polyethylene glycol (*black wavy lines*) to decrease the nonspecific adsorption of the labeled micelles. A 532 nm laser was used to excite the Alexa Fluor 555 fluorophore. This image was prepared with BioRender. *B*, a representative TIRF field-of-view image with the donor channel on the *left* and the acceptor channel on the *right*. Scale bar, 5 μm. ECD, extracellular domain; FRET, fluorescence resonance energy transfer; GCGR, glucagon receptor.

**Figure 2:**
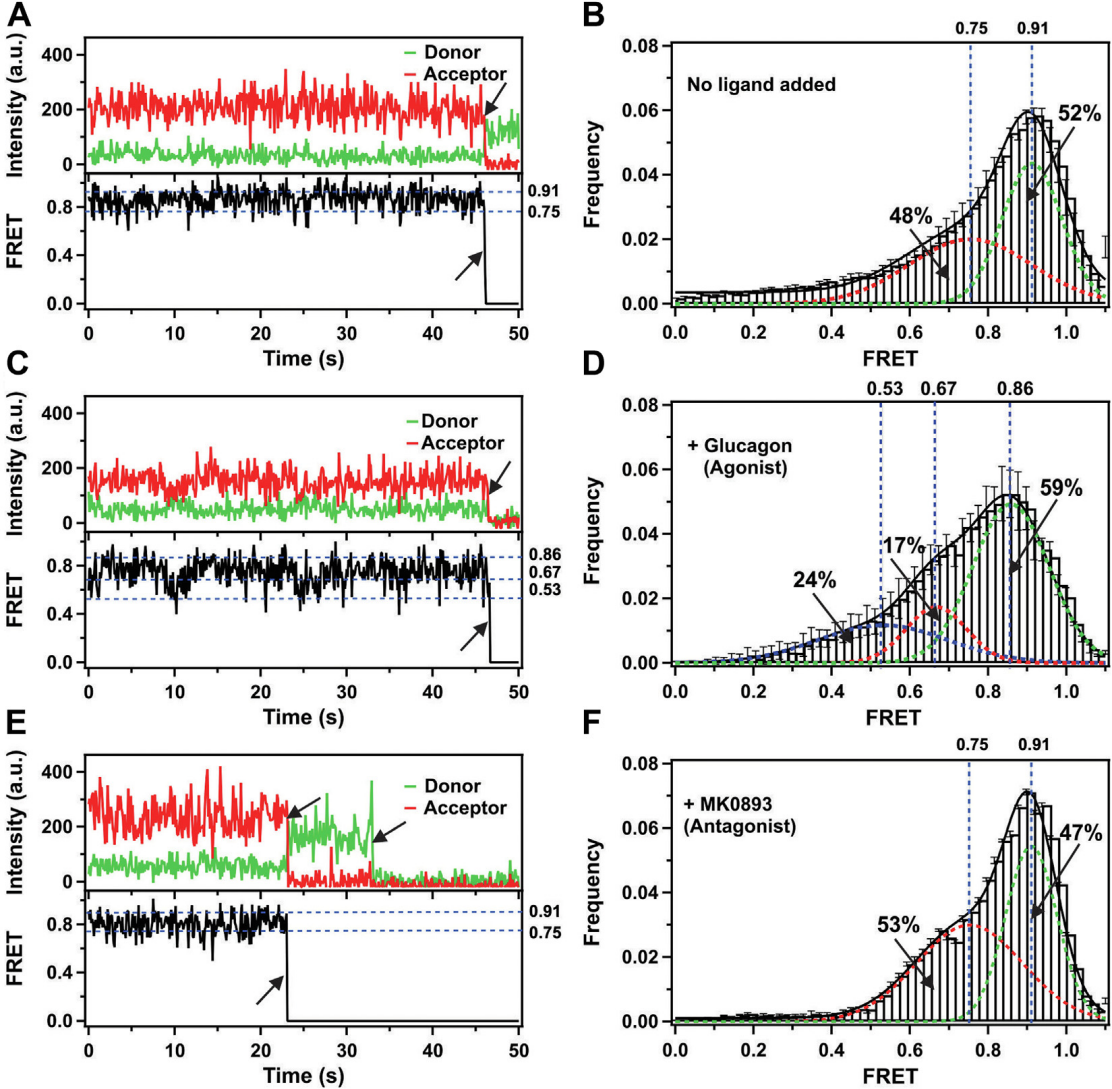
**Representative single-molecule fluorescence trajectories and histograms.***A*, single–molecule intensity time trajectories (*top*: *donor green*; *acceptor red*) and FRET efficiency (*bottom*) for individual apo-GCGR. *Black arrows* represent single-step photobleaching events. *B*, smFRET population histogram compiled from 94 apo-GCGR molecules. The *dashed red* and *green lines* represent two Gaussian populations at positions around 0.75 and 0.91 FRET states. The *solid black line* is the composite fit that represents the sum of the two Gaussians. The percentage populations enclosed by the respective peaks are indicated. Error bars represent the standard errors calculated for each bar. *C*, single-molecule time traces of GCGR in the presence of agonist (glucagon). Other presentation details are the same as in panel *A*. *D*, smFRET population histogram compiled from 64 glucagon-bound GCGR molecules. The *dashed blue*, *red*, and *green lines* represent three Gaussian populations at positions around 0.53, 0.67, and 0.86 FRET states. The *solid black line* is the composite fit that represents the sum of the three Gaussians. *E*, single-molecule time traces of GCGR in complex with the antagonist MK0893. Other presentation details are the same as in panel *A*. *F*, smFRET population histogram compiled from 86 antagonist (MK0893)-bound GCGR molecules. The *dashed red* and *green lines* represent two Gaussian populations at positions around 0.75 and 0.91 FRET states. The *solid black line* is the composite fit that represents the sum of the two Gaussians. FRET, fluorescence resonance energy transfer; GCGR, glucagon receptor; smFRET, single-molecule FRET.

We used smFRET to examine the conformational behavior of the GCGR ECD. smFRET is a well-established technique for studying the conformational dynamics of biomolecules at the single molecule level (38, 39), as it is sensitive to changes in distances *in vitro* and in cells (40). First, we specifically expressed and purified a GCGR with two surface-exposed cysteines at positions 49 (Cys49) of the ECD and 287 (Cys287) of the extracellular side of helix IV (Fig. S4*B*). These two cysteines were site-specifically labeled with a FRET donor (Alexa Fluor 555) and a FRET acceptor (Alexa Fluor 647). The efficiency of energy transfer will report the proximity between the donor and acceptor fluorophores and any changes in that distance (41, 42). To view receptors under smFRET, the doubly labeled GCGR was surface-immobilized on a PEG-passivated quartz microscope slide using a biotin–streptavidin interaction, as shown in Figure 1*A*. A biotinylated anti-FLAG antibody was used to capture the FLAG-tagged GCGR (Fig. 1*A*). A 532 nm laser was used to excite the donor fluorophore by creating the evanescent field from the total internal reflection of the laser beam (Fig. 1*A*). The emission signals from the donor and acceptor fluorophores were detected by an electron-multiplied back-illuminated CCD camera. As shown in Figure 1*B*, individual GCGR molecules are seen in donor and acceptor channels. A control experiment was performed to test the specific immobilization of GCGR–micelle using a biotinylated antibody to a PEG-passivated quartz surface (Fig. S5*A*). We only observed limited receptor molecules without FLAG-tagged antibody on the slide surface, confirming that the GCGR was specifically pulled down by biotinylated anti-FLAG antibody as intended (Fig. S5*B*). Single-molecule fluorescence time trajectories were assessed and analyzed using custom software written in MATLAB. Individual trajectories that showed a single donor and a single acceptor bleaching step (shown by *arrows*) and anticorrelated donor (*green*) and acceptor (*red*) intensity fluctuations (Fig. 2*A*, upper panel, and Fig. S6) were selected for analysis. The corresponding FRET efficiency trajectory (Fig. 2*A*, lower panel) reveals abrupt transitions between two FRET states. This indicates that GCGR ECD transitions between two conformational states in the absence of bound ligands. A FRET histogram compiled from 94 individual molecules revealed two distinct FRET distributions at peaks centered at 0.75 and 0.91 FRET efficiencies, with relative peak areas of 48 and 52%, respectively (Fig. 2*B* and Table S1). On the basis of these observed FRET values, we calculate that the apparent distance between the two fluorophores are around 42.5 Å and 31.0 Å, respectively. These distances are consistent with the measured distances for the open (42.4 Å) and closed (31.9 Å) conformation of the ECD for GCGR and its close homolog GLP-1 (Table S2).

### Glucagon-bound GCGR ECD is dynamic

To study the effect of ligands on GCGR ECD dynamics, we performed similar smFRET measurements in the presence of agonist and antagonist ligands. In the presence of the agonist glucagon (unlabeled), the ECD fluctuated among three FRET states (Figs. 2*C* and S7). A smFRET histogram compiled from 64 individual FRET trajectories revealed at least three FRET distributions with peaks centered at 0.53, 0.67, and 0.86 FRET efficiencies after fitting with three Gaussian functions (Fig. 2*D*). The relative peak areas for the three distributions were calculated as 24%, 17%, and 59% (Fig. 2*D* and Table S1). The center of the high FRET state for the glucagon-bound GCGR shifted to 0.86 from 0.91 FRET state as compared to apo-GCGR. This lower FRET value suggests that glucagon binding triggers a partial opening of the ECD, bringing the donor and acceptor fluorophores further apart, which correlates with the increased distance between the two fluorophores (37.7 Å). This new position of the ECD is not currently observed among currently available class B GPCR structures.

Interestingly, in the presence of glucagon, the ECD remained dynamic, but the FRET states were shifted to lower FRET values as new FRET peaks centered at 0.53 and 0.67 (Fig. 2*D*). These new FRET values correlate with multiple conformations (multiple open states) of the ECD, as proposed previously (26). As a control experiment, we repeated smFRET experiments in the presence of saturating concentration of the GCGR antagonist MK0893 (43). The majority of GCGR molecules in the complex with MK0893 are dynamic and similar to apo-GCGR, as presented in Figure. 2*E* and Fig. S8. The resulting histogram (Fig. 2*F*) compiled from 86 individual molecules shows no effect on the FRET distribution relative to the apo-GCGR, as shown in Figure 2*B* and Table S1. Taken together, these results suggest that the ECD is more open in the presence of glucagon but still shows dynamic behavior (Fig. S9, compare *red* with *black* and *green*).

## Discussion

Single-molecule fluorescence spectroscopy has previously been used to study the conformational dynamics and activation of other GPCRs. Since smFRET largely overcomes ensemble-averaging and time-averaging, it allows the uncovering of individual species in heterogeneous and dynamic biomolecular complexes and also helps to capture transient intermediates (39). Here, we have used smFRET to characterize the dynamic behavior of the ECD of the glucagon receptor. We successfully expressed GCGR in HEK293T cells and purified them using DDM–CHS micelles. The purified receptor retained ligand binding affinity for agonist glucagon as measured by ensemble ligand-binding assay (Fig. S3*B*).

We observed that a single receptor molecule transitioned between two FRET states without any ligands, indicating that the ECD is dynamic (Fig. 2*A*). The corresponding histogram obtained from analyzing many single-molecule trajectories clearly shows two distinct FRET populations around higher (0.91) and lower (0.75) FRET efficiency states (Fig. 2*B*). The higher FRET state corresponds to the ECD being closed, and the lower FRET state to where the ECD is opened. The observation of two FRET states is consistent with previous structural studies that suggest that ECD adopts open and closed conformations (23, 24). The results show that the GCGR ECD is intrinsically dynamic, spending more time in the closed state, but accessing an open state even without an agonist. This is the first report that presents the spontaneous opening and closing of the ECD of any single class B GPCR in real-time. The observation of a lower (0.86 state) FRET state in the presence of a saturating amount of the peptide ligand glucagon indicates a partial opening of the ECD. This observation is consistent with a two-domain binding model in which the C terminus of the peptide ligand first interacts with ECD that triggers the insertion of the N terminus into the orthosteric pocket of the 7TM. Consistent with this model, our data show the appearance of two new low FRET states populated at around 0.67 and 0.53 (Fig. 2, *C* and *D*) for glucagon-bound GCGR. The 0.53 FRET state may correspond to a fully open conformation of the ECD that allows the glucagon peptide to insert into the orthosteric pocket. The calculated distances based on these FRET values are 45.3 Å and 50.0 Å, respectively. These distances agree with ECD open conformation distances measured for several glucagon and antibody-bound GCGR and GLP-1 structures, as summarized in Table S2. Our data suggests that glucagon binding alone is not able to stabilize the ECD at a particular open state, which was not previously reported. Similar to the apo-GCGR, the extrahelical binding antagonist MK0893 does not change the opening and closing states of the ECD. This is expected because this antagonist binds to an allosteric site located outside the 7TM bundle in a position within the TM6-TM7 cleft (43).

**Figure 3:**
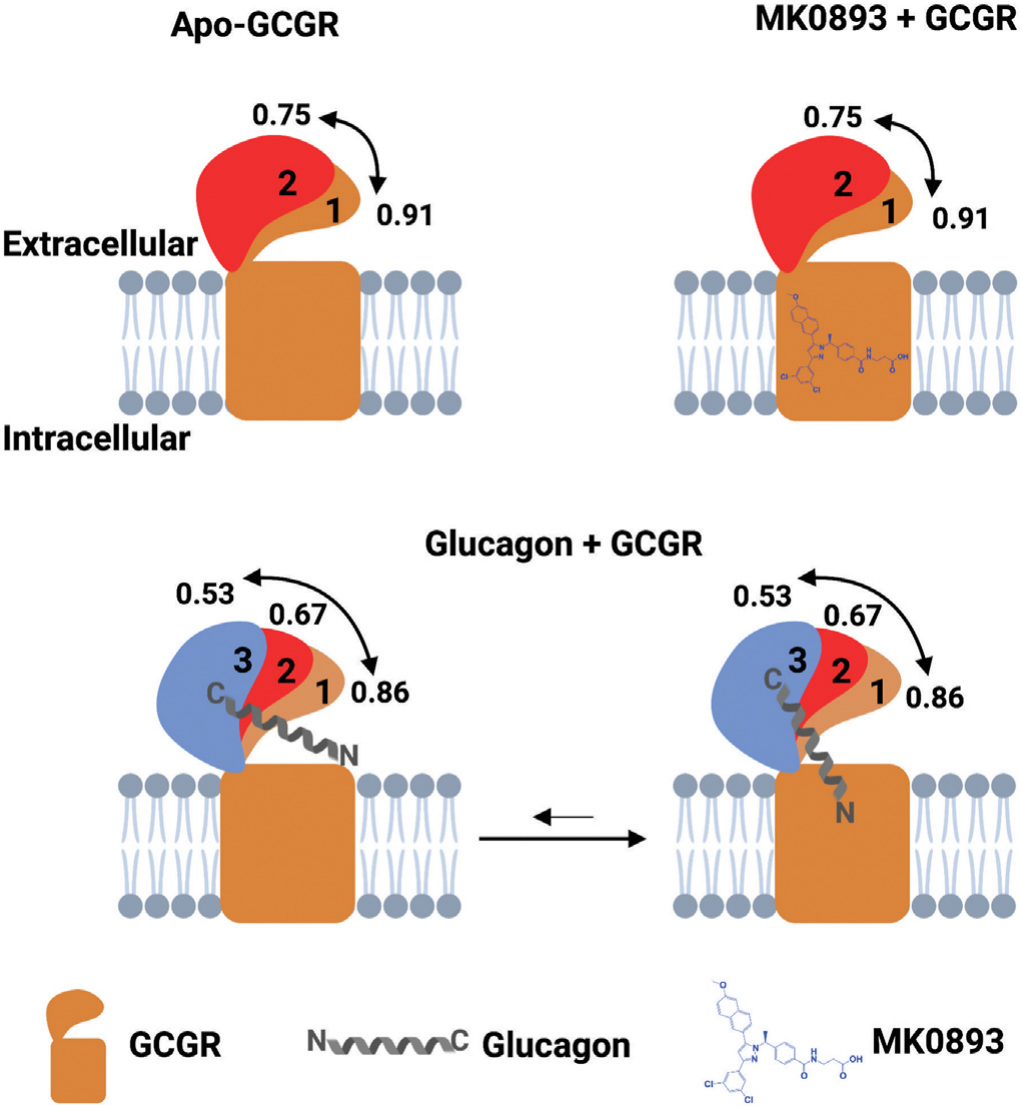
**Schematic of the opening and closing of GCGR ECD generated based on the smFRET data.** In the cartoon, numbers 1, 2, and 3 represent the different positions of ECD drawn based on the number of FRET states populated, as shown in Figure 2. For each ECD position representation, the color of the ECD corresponds to the color of the fitted Gaussian distribution in Figure 2. The *solid double-headed arrows* represent the movement of ECD between the high and low FRET states. For apo (*upper left*) and antagonist MK0893 (*upper right*)-bound GCGR, the ECD transition between close (*green*) and open (*red*) conformations with favorable closed conformation. The binding of the glucagon C terminus to the ECD (*lower left*) triggers a partial opening of the ECD (*green*) that allows the glucagon N terminus to interact with the orthostatic pocket (*lower right*). The *gray* lipid bilayer represents the cell membranes. This image was prepared with BioRender. ECD, extracellular domain; FRET, fluorescence resonance energy transfer; GCGR, glucagon receptor; smFRET, single-molecule FRET.

Our results based on the smFRET data show several key points for the dynamics of class B GPCRs that are illustrated in our working model (Fig. 3). For the first time, we are able to observe the spontaneous opening and closing of the ECD at the molecular level by using site-specifically labeled GCGR. Glucagon binding partially opens the receptor, allowing the N terminus of the glucagon to interact with the receptor core, but it cannot stabilize the ECD at that particular state. The ECD still fluctuates to lower FRET states, which means there is room for dynamic movement of ECD under our experimental conditions. In summary, without agonist binding, the ECD of GCGR naturally fluctuates between open and closed confirmations. Upon glucagon binding, the ECD moves away from the 7TM, making the peptide ligand binding pocket more exposed and potentially allowing insertion of the peptide (Fig. 3).

This study resolved different conformational states of the ECD that enabled us to delineate general mechanisms of ECD dynamics and ligand recognition, an essential step in understanding class B GPCR activation and signaling. Moreover, we can now use these advances to explore how the lipid environment and the presence of G proteins influence ECD dynamics and receptor activation *in vitro* and in the cellular environment. Our approach has the potential to impact not only GCGR but also the study of other class B GPCRs as well. Moreover, these findings may be used in the development of drugs for the treatment of diabetes, subsequently leading to new strategies to stop the progression and dysregulation of high blood glucose.

## Experimental procedures

### Materials

The construct pcDNA3.1(+) GCGR was purchased from GenScript gene synthesis services. The codon-optimized human *GCGR* gene was cloned into a pcDNA3.1(+) vector with a 6xHis tag and a FLAG tag at the C terminus. The accuracy of the construct was confirmed by Sanger sequencing (Center for Environmental Biotechnology, the University of Tennessee). We introduced three mutations (L49C, C171A, and C401A) to the GCGR construct to enable site-specific labeling through thiol-based chemistry. The native cysteine residues C171 and C401 in the TMDs are not involved in disulfide bonding. These modifications did not affect ligand binding or downstream signaling (37, 44). The following items were purchased from Fisher Scientific: Dulbecco's Modified Eagle Medium (#11-320-033), Gibco Opti-MEM I Reduced Serum Medium (#31-985-070), 100× Antibiotic–Antimycotic (#15240062), Avanti Polar Lipids POPC (#NC9445333), DDM (#AAJ6686906), and Ni-NTA resin (PI88221). Polyethyleneimine was purchased from Polysciences (#23966-100). Anti-FLAG antibody (#A01429) was purchased from GenScrip., anti-IgG goat anti-mouse antibody from VWR (#102673-328), glucagon peptide was purchased from Bachem (#4033017), and glucagon [^28^Cys] and [^8^Lys – ^9^Cys] conjugated with Cy5 fluorophore were from Peptide 2.0 Inc. MK0893 was purchased from APExBIO (#A3608).

### HEK293T cell culture and transfection

Human embryonic kidney (HEK) 293T cells were used in this study. The cells are routinely cultured in Dulbecco's Modified Eagle Medium containing l-glutamine, glucose, and sodium pyruvate supplemented with 10% (v/v) fetal bovine serum and 1× antibiotic-antimycotic in humidified 5% (v/v) CO_2_ in air at 37 °C. Cells were seeded in the 10-cm dish until ∼80% confluency, then transfected with a mixture of 10 μg of DNA and 30 μl of polyethyleneimine (1 mg/ml) in 1 ml opti-MEM with reduced serum. GCGR-expressing HEK293T cells were used 48 h posttransfection.

### Gel electrophoresis and immunoblotting

HEK293T cells from 2 to 10-cm cell culture plates were washed with an extracellular buffer and detached from the plates by incubating with Ca^2+^-free DPBS (Fisher Scientific) buffer, followed by a gentle pipetting after 48 h of transfection. Cells were then pelleted by centrifugation at 3000*g* and 4 °C for 10 min. The pellet was lysed in 1 ml RIPA buffer consisting of 150 mM NaCl, 50 mM Tris-HCl (pH 8.0), 5 mM EDTA (pH 8.0), 1% Triton X-100, 0.5% sodium deoxycholate, 0.1% SDS and protease inhibitor cocktail (APExBIO Technology). After 1 h of gentle mixing at 4 °C, cells were centrifuged at 20,000*g* and 4 °C for 15 min, and the supernatant was collected. A 30 μl of this solution was mixed with 30 μl of 2× sample buffer, loaded into a 10% gradient nonreducing polyacrylamide gel, and run at 90 V for the first 25 min and 110 V for another 1 h. A 5 μl portion of precision plus protein standard (Fisher Scientific) was used as a ladder. The gel was washed twice in distilled water for 5 min, incubated in SimplyBlue Safe Stain (Invitrogen) for an hour, and washed in distilled water overnight. Western blotting analysis used a primary anti-FLAG antibody (at 1:1000 dilution) and an anti-IgG goat anti-mouse secondary antibody (at 1:5000 dilution). Gel and membrane were visualized using Odyssey DLx (LI-COR) imaging system.

### Isolation of membrane fractions and DDM solubilization of GCGR

HEK293T cells transiently transfected with GCGR, 48 h after transfection, were washed with ice-cold PBS and harvested in a hypotonic buffer (10 mM Tris pH 7.4 and 4 mM EDTA) with a complete protease inhibitor cocktail. Then, the cells were lysed and homogenized by passing through a 26-gauge syringe 10 to 20 times. The homogenates were centrifuged at 1500*g* for 10 min at 4 °C. The resulting pellets from one 10-cm plate were resuspended in 300 μl of solution A (0.25 M sucrose, 10 mM Tris pH 7.4, and 1 mM EDTA with a complete protease inhibitor cocktail), then mixed thoroughly with 600 μl solution B (2 M sucrose, 10 mM Tris-HCl, pH 7.4, and 1 mM EDTA a containing protease inhibitor cocktail). Finally, the mixture was layered with 200 μl solution A and centrifuged at 30,000*g* for 1 h. The membrane-enriched pellets were collected at the interface between the two sucrose solutions and then resuspended in a hypotonic buffer. The suspension was centrifuged again at 30,000*g* for 30 min resulting in the separation of membrane pellets. GCGR-containing membranes were incubated with solubilization buffer (20 mM HEPES, 100 mM NaCl, 5 mM MgCl_2,_ 0.05% (w/v) DDM, 0.01% (w/v) CHS (Anatrace #D310-CH210), plus a Complete EDTA-free protease inhibitor cocktail) for 3 to 4 h at 4 °C.

### Ni^2+^–NTA affinity purification

The GCGR micelle with 6xHis tag was incubated with Ni-NTA resin pre-equilibrated with solubilization buffer with 10 mM imidazole and allowed gentle rotation overnight at 4 °C. Solubilization buffer with 25 mM imidazole was used to wash the column 15 to 20 times. The GCGR micelle was then eluted with 1 ml solubilization buffer containing 250 mM imidazole and were collected in 0.2 ml fractions in collected in individually labeled Eppendorf tubes.

### Fluorophore conjugation of GCGR-micelle

GCGR micelles (L49C and C287) were incubated with a 5-fold molar excess of Alexa Fluor 555 maleimide (Fluoroprobes #1168) and 10-fold molar excess of Alexa Fluor 647 maleimide (Fluoroprobes #1122) at 4 °C for 3 to 4 h with gentle rotation. To remove excess fluorophores and concentrate the protein, the mixture was applied in the Amicon Ultra centrifugal filters (Millipore #UFC501096) and centrifuged at 10,000*g* for 20 min. Wash buffer (20 mM HEPES pH 7.4, 100 mM NaCl, 5 mM MgCl_2,_ and 0.05% DDM 0.01% CHS) was added to the filter to centrifuge three times at 10,000*g* for 20 min each time.

### Mass photometry

A mass photometry experiment was performed as described previously (45, 46). Microscope cover glasses (No. 1.5H, 24 × 50 mm, Deckgläser) were cleaned by consecutive sonication in Milli-Q water, isopropanol, and Milli-Q water, followed by drying with a clean nitrogen stream. GCGR micelles (diluted 100 times with filtered 1× PBS) were added to the reusable gaskets (3 mm DIA × 1 mm Depth, GBL103250, Grace Bio-Labs) on the microscope coverslips. Data were acquired using AcquireMP (Refeyn Ltd, version 2022 R1) on a One^MP^ mass photometer (Refeyn Ltd). For each acquisition, 18 μl of diluted GCGR-micelle was added, followed by autofocus stabilization and recording of 60 s movies. Each measurement was performed at least three independent times (n ≥ 3). The calibration curve was performed using standards: thymoglobulin (MW 660 kDa), apoferritin (MW 480 kDa), and BSA (MW 66 kDa/132 kDa). Thymoglobulin, apoferritin, and BSA were resuspended in 250 μl, 625 μl, and 750 μl of filtered 1× PBS buffer, respectively. All mass photometry images were processed and analyzed using DiscoverMP (Refeyn Ltd, version 2022 R1).

### Quantification and analysis of ligand binding for GCGR in micelle

The Agilent Cary Eclipse Fluorescence Spectrophotometer was used to perform the interaction of the ligand with GCGR. In this experiment, 100 nM GCGR labeled with Alexa Fluor 555 was present, and the concentration of glucagon labeled with Alexa Fluor 647 was varied by starting at a low concentration (1 nM) and followed by gradually increasing concentration until it reached to the highest (10 μM). Calculated FRET efficiency and varied glucagon concentrations were plotted using Igor Pro software. The plot was fitted with the Hill equation.

### Total internal reflection fluorescence–based smFRET experiments

Single-molecule fluorescence data were recorded using a customized prism-based total internal reflection fluorescence imaging system based on an inverted IX73 microscope (Olympus) equipped with a X60 objective (Olympus, 1.49 numerical aperture, water) as described in earlier publications (47, 48, 49). Quartz microscope slides (G. Finkenbeiner) and coverslips (Fisher Scientific #1254588) were cleaned and passivated with polyethylene glycol (m-PEG-SVA) and 3% (w/w) biotin-PEG-SVA (Laysan Bio) to prevent nonspecific protein adsorption, as previously described (30). A sample chamber was prepared using double-sided tape (Scotch) on PEGylated slides and coverslips (48). The sample chamber was first coated with 0.02 mg/ml NeutrAvidin (ThermoFisher Scientific #PI31000) and washed with T50 buffer (10 mM Tris, pH 7.4, 50 mM NaCl). A 20 μM biotin-conjugated anti-FLAG antibody (GenScript #A01429) was injected and incubated for 20 min, followed by washing with the imaging buffer (25 mM HEPES pH 7.5, 150 mM NaCl, 2 mM Trolox, 0.05% DDM, 0.01% CHS) to remove unbound biotinylated anti-FLAG antibody. The GCGR sample was diluted in an imaging buffer, introduced into the sample chamber, and incubated for 30 min. The sample chamber was washed extensively with an imaging buffer to remove any unbound receptors. Finally, an oxygen scavenging system, 5 mM protocatechuic acid (Sigma #37580), and 3 U/ml protocatechuate-3,4-dioxygenase (Oriental Yeast) (50) in the imaging buffer were applied before total internal reflection fluorescence illumination. The sample was excited at 532 nM (CrystaLaser), and emission intensities from both donor and acceptor fluorophores were collected on an EMCCD camera (Andor Technology) with 100 ms integration time using a custom single-molecule data acquisition program (48). All single-molecule measurements were performed at room temperature. Single-molecule time trajectories were extracted using scripts written in IDL software (Harris Geospatial Solutions, Inc). The data acquisition and extraction package were downloaded from Dr Taekjip Ha's laboratory (http://ha.med.jhmi.edu/resources/).

### Analysis of smFRET data

smFRET data analysis was described as previously (47, 51). Briefly, single-molecule fluorescence time trajectories were assessed and analyzed using custom software written in MATLAB. We observed 43% of molecules with a donor only, 44% with a single donor and single acceptor, and 13% labeled with two donors. Individual trajectories with a single donor and acceptor bleaching step during the acquisition time, stable total intensity ($I_{D}+I_{A}$), and anticorrelated donor and acceptor intensities without blinking events that lasted for more than 5 s were manually selected for further analysis. Individual dynamic traces were then baseline corrected by using the mean intensity levels in each donor and acceptor traces after photobleaching. FRET efficiency was calculated using the equation, $E_{FRET}=I_{A}/\left(I_{D}+I_{A}\right)$, where *I*_D_ and *I*_A_ are corrected donor and acceptor intensities, respectively. Each FRET trace was truncated before photobleaching, and the traces were binned and compiled to generate FRET histograms. All experiments were repeated at least three times independently to ensure the reproducibility of the results. Unless otherwise stated, the histograms for each GCGR complex were generated by compiling at least 100 FRET traces from individual molecules. Error bars on the histogram are the standard error calculated from at least five independent movies used to generate the histograms (34, 49). FRET populations on histograms were fit to Gaussian distributions using the Multipeak Fit 2 function in the Igor Pro software (version 8.04, Wavemetrics) as described previously (49, 52). In more detail, we used “The Find Peaks” (Igor Pro) algorithm to locate the number of peaks and fit those peaks using the mean conformational states as peak centers identified by the program. The program only found two peaks for apo and MK0893 bound and three peaks for glucagon bound samples. We then used Igor’s Multipeak Fit 2 function, $y\left(x\right)=y_{0}+\sum_{i=1}^{3}A_{i}\cdot e^{{-\left(\frac{x-\mu_{i}}{\sigma_{i}}\right)}^{2}}$ to fit the Gaussian distributions. In this equation, $y_{0}$ is the baseline, i is the number of the gaussian peaks, $A_{i}$ is the height, $\mu_{i}$ is the location, and $\sigma_{i}$ is the width of the corresponding gaussian peak. We tested the goodness of the fit by looking at the chi-square analysis. The area of individual peaks was calculated from the $A_{i}$ and $\sigma_{i}$ values obtained from the fit.

The peak centers observed from the fitted histograms were used to calculate the distance between two dyes using the following Förster equation.

$$E_{FRET}=\frac{1}{1+{\left(\frac{R}{R_{0}}\right)}^{6}}$$

Where *R* is the distance and *R*_*0*_ is the Förster distance between two dyes. We used *R*_*0*_ value of 51 Å for Alexa Fluor 555 and Alexa Fluor 647 dye-pair to calculate distances.

## Data availability

All data reported in this manuscript will be shared by the lead contact upon request.

## Supporting information

This article contains supporting information.

## Conflict of interest

The authors declare that they have no conflicts of interest with the contents of this article.

## References

[1] Wang M.Y., Yan H., Shi Z., Evans M.R., Yu X., Lee Y. (2015). Glucagon receptor antibody completely suppresses type 1 diabetes phenotype without insulin by disrupting a novel diabetogenic pathway. Proc. Natl. Acad. Sci. U. S. A..

[2] Lee Y.H., Wang M.Y., Yu X.X., Unger R.H. (2016). Glucagon is the key factor in the development of diabetes. Diabetologia.

[3] Steenberg V.R., Jensen S.M., Pedersen J., Madsen A.N., Windelov J.A., Holst B. (2016). Acute disruption of glucagon secretion or action does not improve glucose tolerance in an insulin-deficient mouse model of diabetes. Diabetologia.

[4] Damond N., Thorel F., Moyers J.S., Charron M.J., Vuguin P.M., Powers A.C. (2016). Blockade of glucagon signaling prevents or reverses diabetes onset only if residual beta-cells persist. Elife.

[5] Neumann U.H., Ho J.S.S., Mojibian M., Covey S.D., Charron M.J., Kieffer T.J. (2016). Glucagon receptor gene deletion in insulin knockout mice modestly reduces blood glucose and ketones but does not promote survival. Mol. Metab..

[6] Liang Y., Osborne M.C., Monia B.P., Bhanot S., Gaarde W.A., Reed C. (2004). Reduction in glucagon receptor expression by an antisense oligonucleotide ameliorates diabetic syndrome in db/db mice. Diabetes.

[7] Sorensen H., Brand C.L., Neschen S., Holst J.J., Fosgerau K., Nishimura E. (2006). Immunoneutralization of endogenous glucagon reduces hepatic glucose output and improves long-term glycemic control in diabetic ob/ob mice. Diabetes.

[8] Petersen K.F., Sullivan J.T. (2001). Effects of a novel glucagon receptor antagonist (Bay 27-9955) on glucagon-stimulated glucose production in humans. Diabetologia.

[9] Shah P., Basu A., Basu R., Rizza R. (1999). Impact of lack of suppression of glucagon on glucose tolerance in humans. Am. J. Physiol..

[10] Shah P., Vella A., Basu A., Basu R., Schwenk W.F., Rizza R.A. (2000). Lack of suppression of glucagon contributes to postprandial hyperglycemia in subjects with type 2 diabetes mellitus. J. Clin. Endocrinol. Metab..

[11] Qureshi S.A., Rios Candelore M., Xie D., Yang X., Tota L.M., Ding V.D. (2004). A novel glucagon receptor antagonist inhibits glucagon-mediated biological effects. Diabetes.

[12] Djuric S.W., Grihalde N., Lin C.W. (2002). Glucagon receptor antagonists for the treatment of type II diabetes: current prospects. Curr. Opin. Investig. Drugs.

[13] Sloop K.W., Cao J.X., Siesky A.M., Zhang H.Y., Bodenmiller D.M., Cox A.L. (2004). Hepatic and glucagon-like peptide-1-mediated reversal of diabetes by glucagon receptor antisense oligonucleotide inhibitors. J. Clin. Invest..

[14] Koth C.M., Murray J.M., Mukund S., Madjidi A., Minn A., Clarke H.J. (2012). Molecular basis for negative regulation of the glucagon receptor. Proc. Natl. Acad. Sci. U. S. A..

[15] Ye L., Van Eps N., Zimmer M., Ernst O.P., Prosser R.S. (2016). Activation of the A2A adenosine G-protein-coupled receptor by conformational selection. Nature.

[16] Vafabakhsh R., Levitz J., Isacoff E.Y. (2015). Conformational dynamics of a class C G-protein-coupled receptor. Nature.

[17] Nygaard R., Zou Y., Dror R.O., Mildorf T.J., Arlow D.H., Manglik A. (2013). The dynamic process of beta(2)-adrenergic receptor activation. Cell.

[18] Wingler L.M., Elgeti M., Hilger D., Latorraca N.R., Lerch M.T., Staus D.P. (2019). Angiotensin analogs with divergent bias stabilize distinct receptor conformations. Cell.

[19] Parthier C., Reedtz-Runge S., Rudolph R., Stubbs M.T. (2009). Passing the baton in class B GPCRs: peptide hormone activation via helix induction?. Trends Biochem. Sci..

[20] Mann R., Wigglesworth M.J., Donnelly D. (2008). Ligand-receptor interactions at the parathyroid hormone receptors: subtype binding selectivity is mediated via an interaction between residue 23 on the ligand and residue 41 on the receptor. Mol. Pharmacol..

[21] Hollenstein K., de Graaf C., Bortolato A., Wang M.W., Marshall F.H., Stevens R.C. (2014). Insights into the structure of class B GPCRs trends. Pharmacol. Sci..

[22] Zhao L.H., Yin Y., Yang D., Liu B., Hou L., Wang X. (2016). Differential requirement of the extracellular domain in activation of class B G protein-coupled receptors. J. Biol. Chem..

[23] Yang L., Yang D., de Graaf C., Moeller A., West G.M., Dharmarajan V. (2015). Conformational states of the full-length glucagon receptor. Nat. Commun..

[24] Zhang H., Qiao A., Yang D., Yang L., Dai A., de Graaf C. (2017). Structure of the full-length glucagon class B G-protein-coupled receptor. Nature.

[25] Zhang H., Qiao A., Yang L., Van Eps N., Frederiksen K.S., Yang D. (2018). Structure of the glucagon receptor in complex with a glucagon analogue. Nature.

[26] Wu F., Yang L., Hang K., Laursen M., Wu L., Han G.W. (2020). Full-length human GLP-1 receptor structure without orthosteric ligands. Nat. Commun..

[27] Zhao L.H., Ma S., Sutkeviciute I., Shen D.D., Zhou X.E., de Waal P.W. (2019). Structure and dynamics of the active human parathyroid hormone receptor-1. Science.

[28] Kahlscheuer M.L., Widom J., Walter N.G. (2015). Single-molecule pull-down FRET to dissect the mechanisms of biomolecular machines. Methods Enzymol..

[29] Lerner E., Barth A., Hendrix J., Ambrose B., Birkedal V., Blanchard S.C. (2021). FRET-based dynamic structural biology: challenges, perspectives and an appeal for open-science practices. Elife.

[30] Lamichhane R., Solem A., Black W., Rueda D. (2010). Single-molecule FRET of protein-nucleic acid and protein-protein complexes: surface passivation and immobilization. Methods.

[31] Juette M.F., Terry D.S., Wasserman M.R., Zhou Z., Altman R.B., Zheng Q. (2014). The bright future of single-molecule fluorescence imaging. Curr. Opin. Chem. Biol..

[32] Lamichhane R., Hammond J.A., Pauszek R.F., Anderson R.M., Pedron I., van der Schans E. (2017). A DEAD-box protein acts through RNA to promote HIV-1 Rev-RRE assembly. Nucleic Acids Res..

[33] Gregorio G.G., Masureel M., Hilger D., Terry D.S., Juette M., Zhao H. (2017). Single-molecule analysis of ligand efficacy in beta(2)AR-G-protein activation. Nature.

[34] Liauw B.W., Afsari H.S., Vafabakhsh R. (2021). Conformational rearrangement during activation of a metabotropic glutamate receptor. Nat. Chem. Biol..

[35] Asher W.B., Geggier P., Holsey M.D., Gilmore G.T., Pati A.K., Meszaros J. (2021). Single-molecule FRET imaging of GPCR dimers in living cells. Nat. Methods.

[36] Kuszak A.J., Pitchiaya S., Anand J.P., Mosberg H.I., Walter N.G., Sunahara R.K. (2009). Purification and functional reconstitution of monomeric mu-opioid receptors: allosteric modulation of agonist binding by Gi2. J. Biol. Chem..

[37] Siu F.Y., He M., de Graaf C., Han G.W., Yang D., Zhang Z. (2013). Structure of the human glucagon class B G-protein-coupled receptor. Nature.

[38] Choi U.B., Strop P., Vrljic M., Chu S., Brunger A.T., Weninger K.R. (2010). Single-molecule FRET-derived model of the synaptotagmin 1-SNARE fusion complex. Nat. Struct. Mol. Biol..

[39] Ha T., Enderle T., Ogletree D.F., Chemla D.S., Selvin P.R., Weiss S. (1996). Probing the interaction between two single molecules: fluorescence resonance energy transfer between a single donor and a single acceptor. Proc. Natl. Acad. Sci. U. S. A..

[40] Sakon J.J., Weninger K.R. (2010). Detecting the conformation of individual proteins in live cells. Nat. Methods.

[41] Ma L., Yang F., Zheng J. (2014). Application of fluorescence resonance energy transfer in protein studies. J. Mol. Struct..

[42] Roy R., Hohng S., Ha T. (2008). A practical guide to single-molecule FRET. Nat. Methods.

[43] Jazayeri A., Dore A.S., Lamb D., Krishnamurthy H., Southall S.M., Baig A.H. (2016). Extra-helical binding site of a glucagon receptor antagonist. Nature.

[44] Hilger D., Kumar K.K., Hu H., Pedersen M.F., O'Brien E.S., Giehm L. (2020). Structural insights into differences in G protein activation by family A and family B GPCRs. Science.

[45] Olerinyova A., Sonn-Segev A., Gault J., Eichmann C., Schimpf J., Kopf A.H. (2021). Mass photometry of membrane proteins. Chem.

[46] Diwanji D., Trenker R., Thaker T.M., Wang F., Agard D.A., Verba K.A. (2022). Author correction: structures of the HER2-HER3-NRG1beta complex reveal a dynamic dimer interface. Nature.

[47] Berezhna S.Y., Gill J.P., Lamichhane R., Millar D.P. (2012). Single-molecule Forster resonance energy transfer reveals an innate fidelity checkpoint in DNA polymerase I. J. Am. Chem. Soc..

[48] Stefanski K.M., Russell C.M., Westerfield J.M., Lamichhane R., Barrera F.N. (2021). PIP2 promotes conformation-specific dimerization of the EphA2 membrane region. J. Biol. Chem..

[49] Wei S., Thakur N., Ray A.P., Jin B., Obeng S., McCurdy C.R. (2022). Slow conformational dynamics of the human A2A adenosine receptor are temporally ordered. Structure.

[50] Aitken C.E., Marshall R.A., Puglisi J.D. (2008). An oxygen scavenging system for improvement of dye stability in single-molecule fluorescence experiments. Biophys. J..

[51] Lamichhane R., Berezhna S.Y., Gill J.P., Van der Schans E., Millar D.P. (2013). Dynamics of site switching in DNA polymerase. J. Am. Chem. Soc..

[52] Thakur N., Wei S., Ray A.P., Lamichhane R., Eddy M.T. (2022). Production of human A(2A)AR in lipid nanodiscs for (19)F-NMR and single-molecule fluorescence spectroscopy. STAR Protoc..

