# Supplementary material for: Single-molecule analysis reveals that a glucagon-bound extracellular domain of the glucagon receptor is dynamic
Source: J Biol Chem. 2023 Aug 14;299(9):105160. doi: 10.1016/j.jbc.2023.105160 (PMC10514447; doi:10.1016/j.jbc.2023.105160)
Supplement: Supporting information [file mmc1.docx]

**Single-molecule analysis reveals that a glucagon-bound extracellular domain of the glucagon receptor is dynamic**

Ting Liu^1^, Susmita Khanal^1^, Gillian D. Hertslet^1^, Rajan Lamichhane^1*^

^1^Department of Biochemistry & Cellular and Molecular Biology, College of Arts & Sciences, University of Tennessee, Knoxville, TN 37932, USA

*For correspondence:

Rajan Lamichhane

University of Tennessee Knoxville

Department of Biochemistry & Cellular and Molecular Biology

1311 Cumberland Ave., Knoxville, TN 37996, USA

Phone: 865-974-0650

Email: [rajan@utk.edu](mailto:rajan@utk.edu)

*Supporting Information (SI)*

Supplemental Figure S1:

Supplemental Figure S2:

Supplemental Figure S3:

Supplemental Figure S4:

Supplemental Figure S5:

Supplemental Figure S6:

Supplemental Figure S7:

Supplemental Figure S8:

Supplemental Figure S9:

Supplemental Table S1:

Supplemental Table S2:

Supporting Text:

Supplemental References:

**Supplemental Figure S1**

**
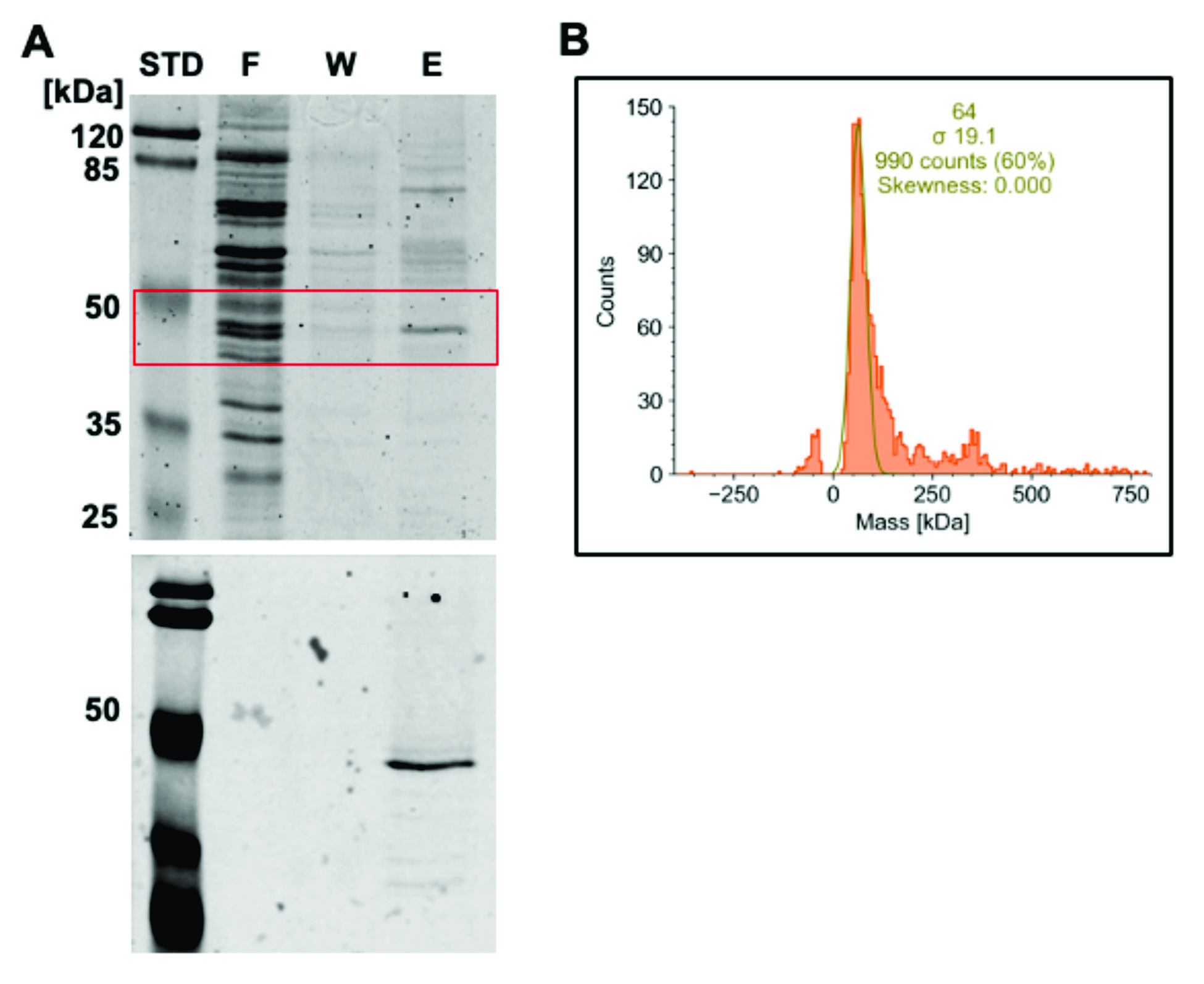
**

**Figure S1. Characterization of GCGR in the micelle.** (A) Coomassie-stained SDS-PAGE (upper panel) and western blot (lower panel) analysis after GCGR purification. The lane contents are as follows: F-Flow through; W-Wash through; E-Elution from Ni-NTA Resin column. The red box denotes the bands corresponding to the GCGR protein. (B) Mass photometry of the peak sample indicates that the majority of particles have an average mass of ~64 kDa with a standard deviation of 19.1 kDa.

**Supplemental Figure S2**

**
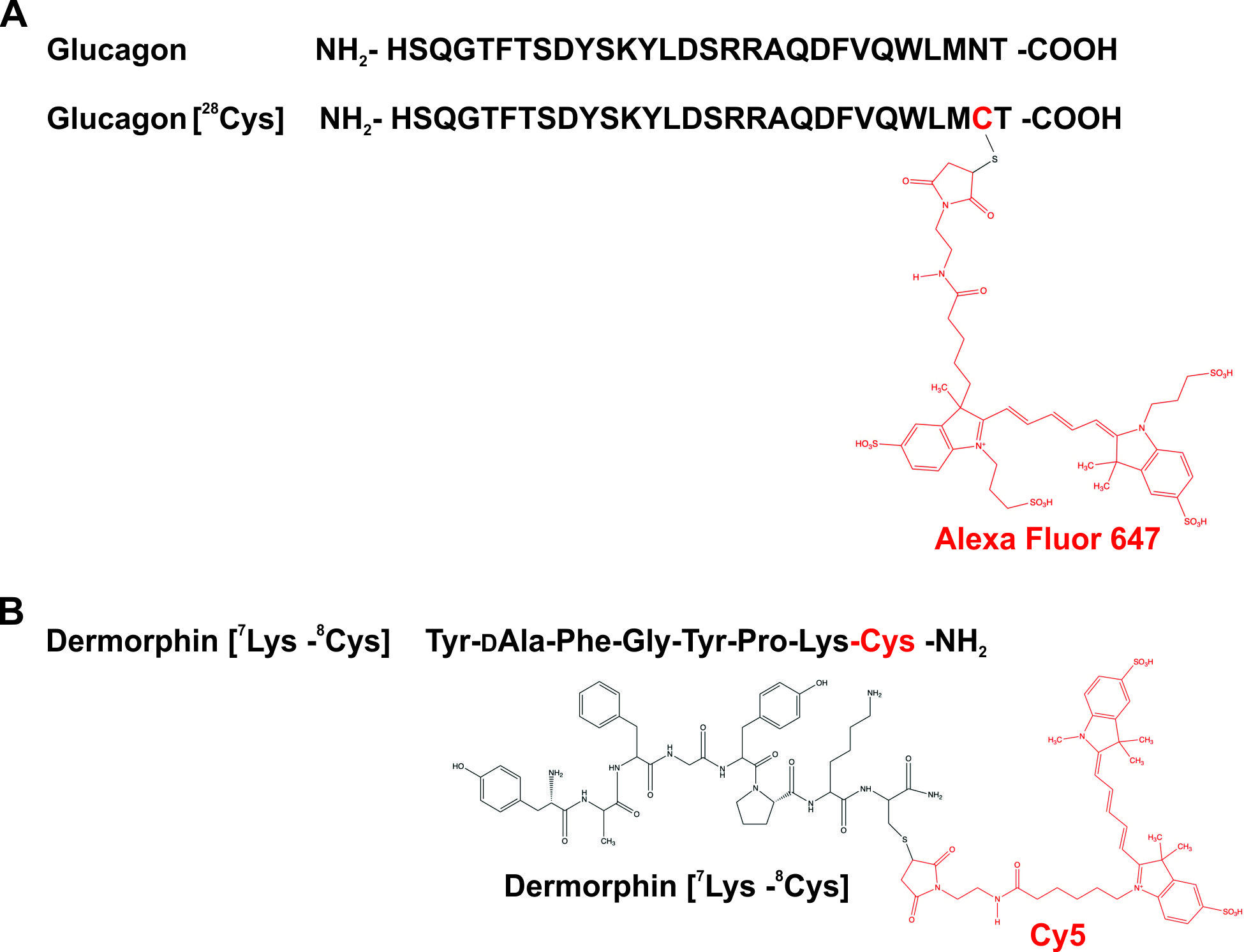
**

**Figure S2. Sequence of peptides used in the study.** (A) Sequence of glucagon and ^28^Cys modified peptides. The ^28^Cys was used to label Alexa Fluor 647 as presented. (B) Sequence of dermorphin [^7^Lys - ^8^Cys] peptide and labeled Cy5 at ^8^Cys.

**Supplementary Figure S3**

**
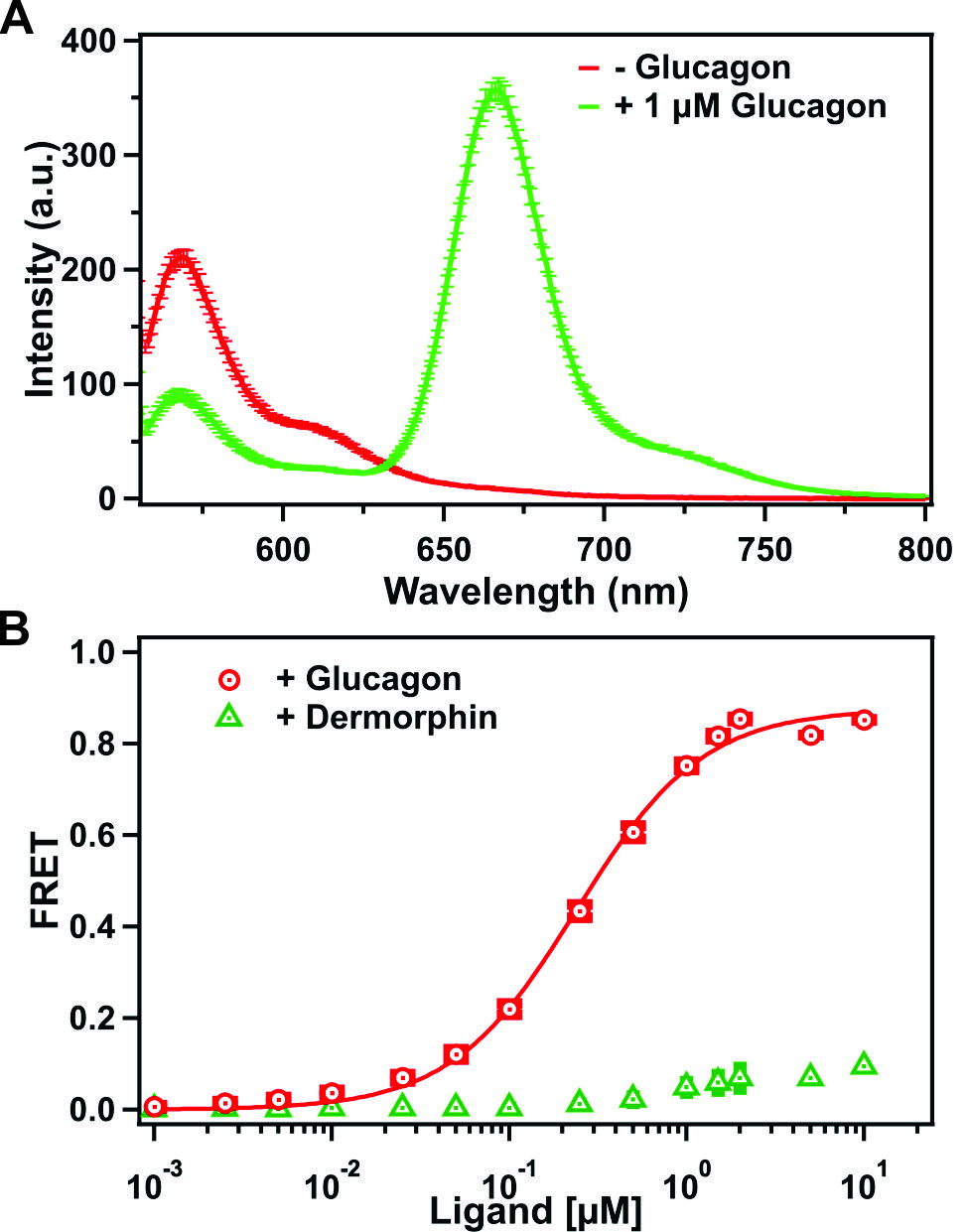
**

**Figure S3. Interaction of GCGR with glucagon.** (A) Fluorescence emission spectra of donor-only labeled GCGR in the absence of glucagon (red) and in the presence of 1 µM acceptor (Alexa Fluor 647) labeled glucagon (green). (B) Calculated FRET efficiency as a function of labeled glucagon (red) and Cy5 labeled dermorphin (green) concentrations. The red line is a fit to a Hill equation (see methods). Data represent mean ± S.E.M. of N=3 independent experiments.

**Supplemental Figure S4**


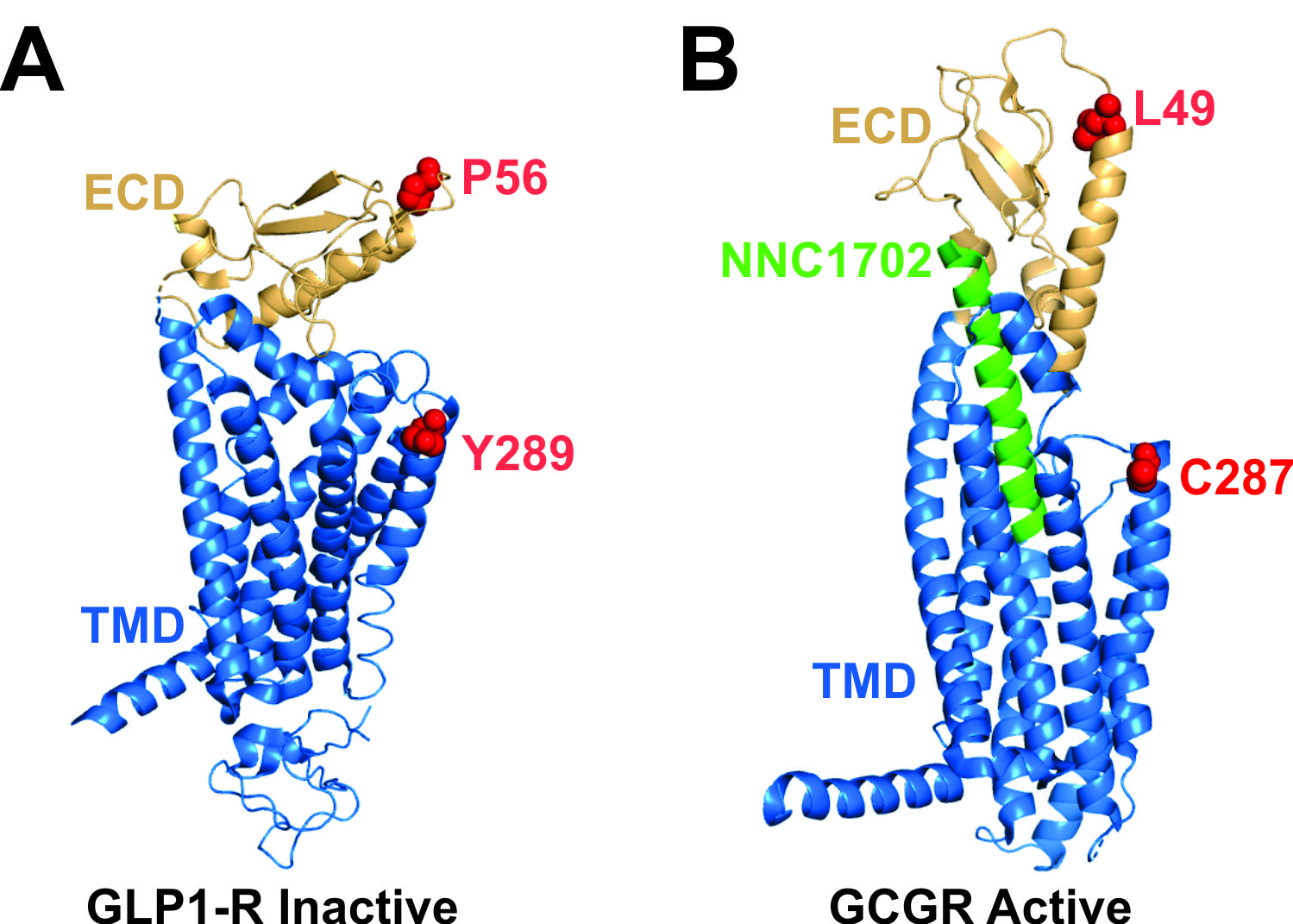


**Figure S4. Structural comparison of open and closed states of GCGR and GLP-1R ECD.** (A) GLP1-R inactive; PDB: 6LN2. (B) GCGR active with bound partial agonist (NNC1702); PBD: 5YQZ. ECD: orange, TMD: blue, Ligand: green, Labeling positions and amino acids: red.

**Supplemental Figure S5**

**
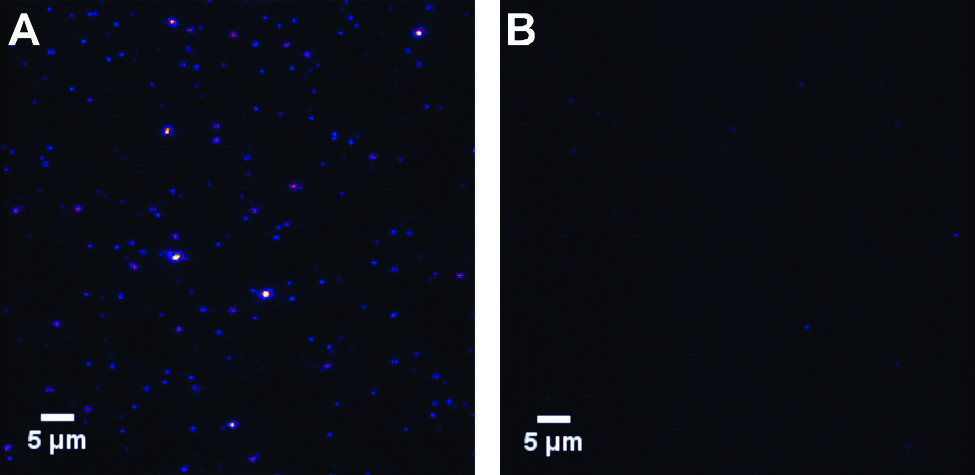
**

**Figure S5. TIRF field-of-view images** (A) GCGR micelles were captured on the slides through a biotinylated anti-FLAG antibody. Each spot represents a single GCGR micelle. The very bright spots were from aggregates excluded from the data analysis. (B) A corresponding TIRF image for a control experiment surface lacking a biotinylated anti-FLAG antibody. The number of fluorescent spots is dramatically reduced relative to A, indicating a negligible level of nonspecific adsorption of GCGR micelles on the PEG passivated slide surface.

**Supplemental Figure S6**

**
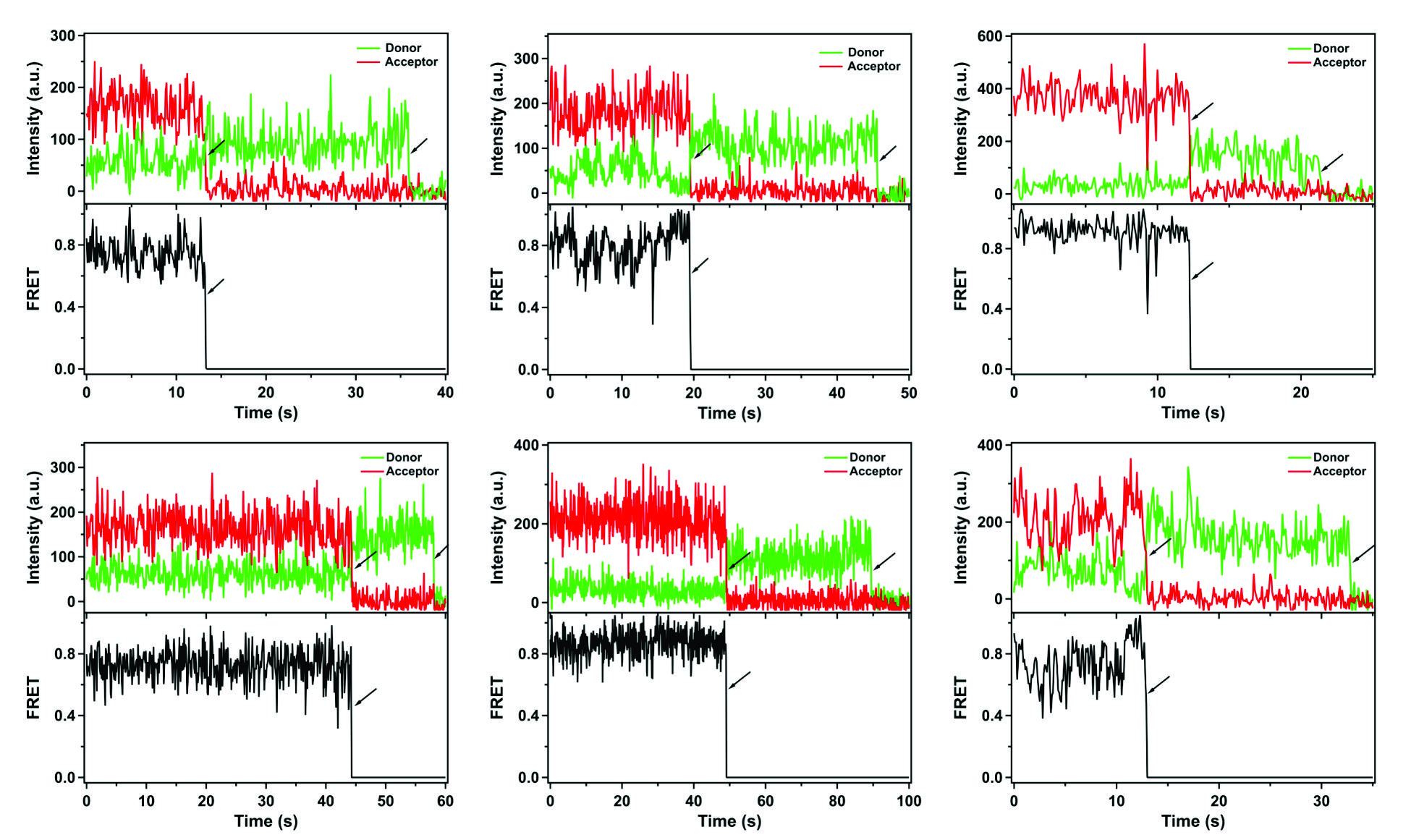
**

**Figure S6.** Example single-molecule time traces of apo-GCGR.

**Supplemental Figure S7**

**
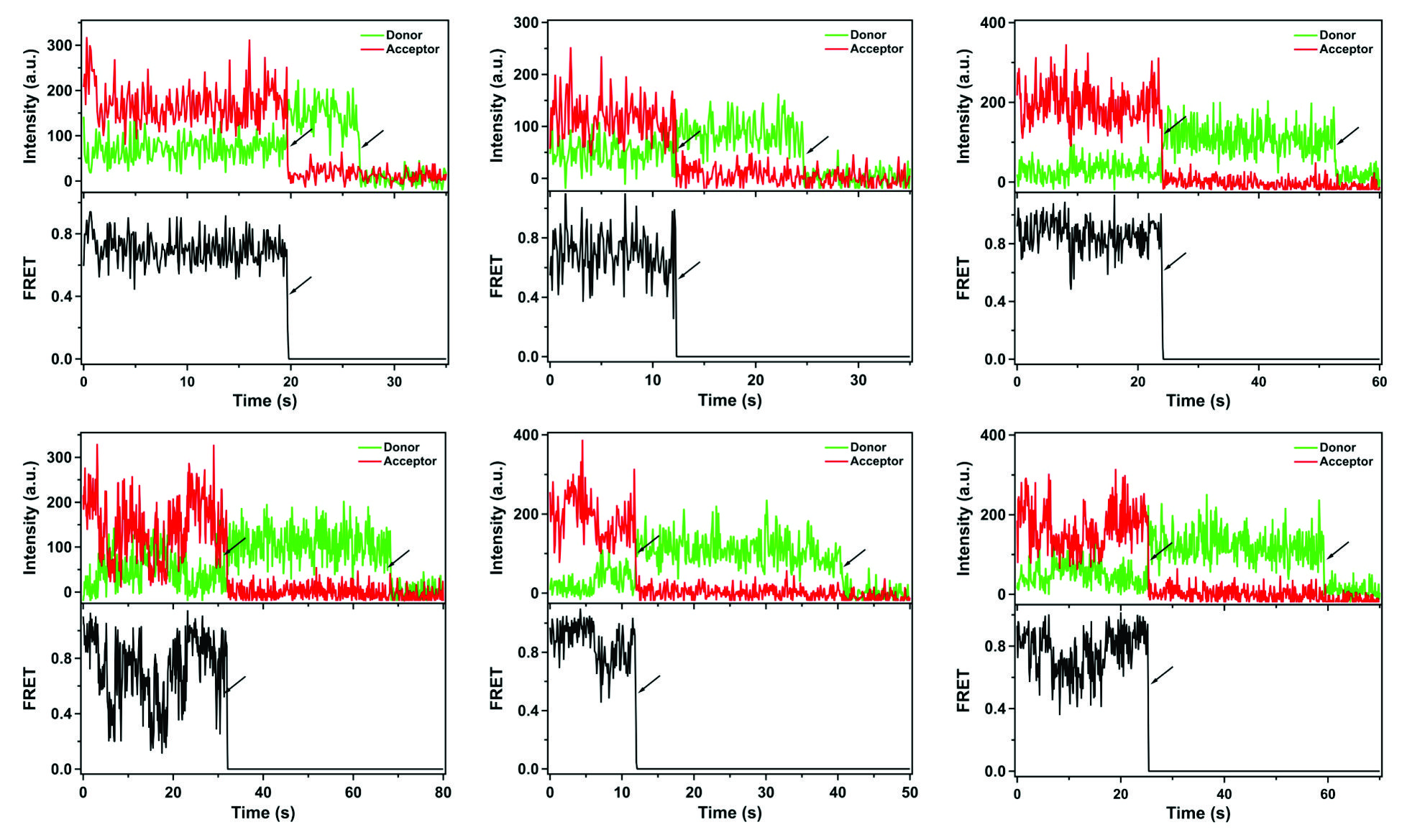
**

**Figure S7.** Example single-molecule time traces of glucagon bound GCGR.

**Supplemental Figure S8**

**
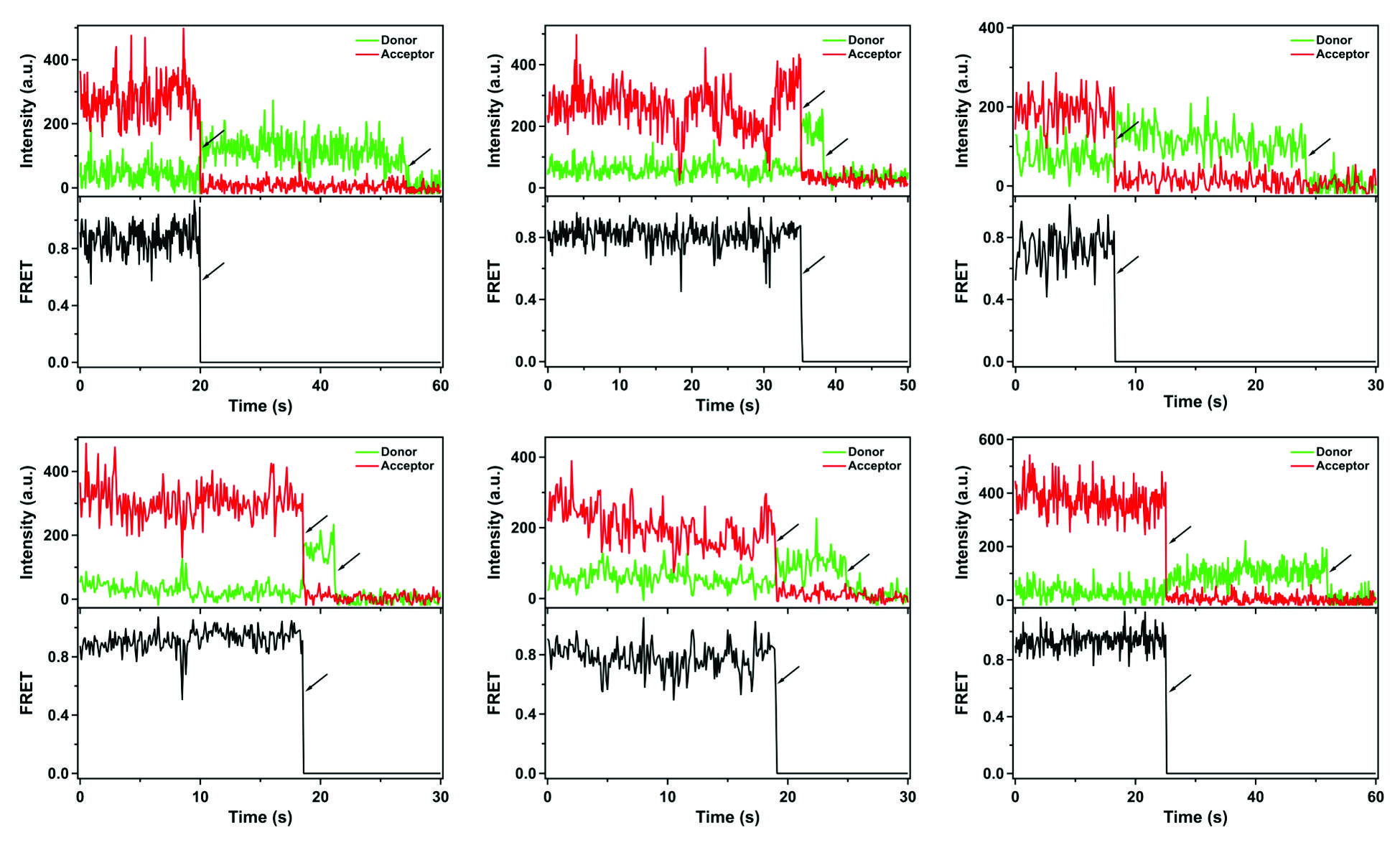
**

**Figure S8.** Example single-molecule time traces of MK0893 bound GCGR.

**Supplemental Figure S9**


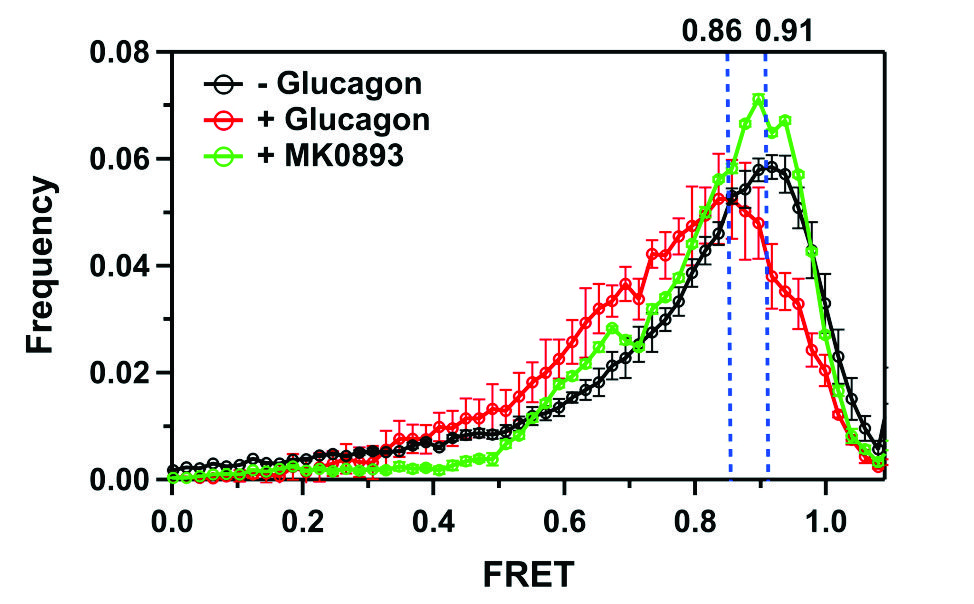


**Figure S9.** Combined smFRET population histograms of apo (black), glucagon bound (red), and MK0893 bound (green) GCGR complexes. The vertical dashed lines represent the highest FRET states observed for different complexes.

**Supplementary Table S1. Single-molecule FRET histogram fit parameters from Figure 2.**

| Peaks | No Ligand Added | | +Glucagon  (Agonist) | | | +MK0893 (Antagonist) | |
| --- | --- | --- | --- | --- | --- | --- | --- |
|  | Peak1 (Red) | Peak 2 (Green) | Peak 1 (Blue) | Peak 2 (Red) | Peak 3 (Green) | Peak 1 (Red) | Peak 1 (Green) |
| Location | 0.75 | 0.91 | 0.53 | 0.67 | 0.86 | 0.75 | 0.91 |
| Width | 0.22 | 0.11 | 0.24 | 0.11 | 0.14 | 0.19 | 0.09 |
| Area | 48% | 52% | 24% | 17% | 59% | 53% | 47% |
| Chi-Square | 2.78 × 10^-4^ | | 7.86 × 10^-5^ | | | 2.16 × 10^-4^ | |

**Supplementary Table S2. Distances distributions taken from published structures for GCGR and its homolog GLP-1.**

| Receptor | PDB File | ^a^ Construct with | Ligand | ^b^Distance Å | Reference |
| --- | --- | --- | --- | --- | --- |
| GLP-1R | 6LN2 | Fab7F38 Fragment | Fab738 | 31.9 | 1 |
| GCGR | 5XEZ | mAB1 Antibody | NNC0640 | 50.3 | 2 |
| GCGR | 5YQZ | Partial Agonist | NNC1702 | 42.4 | 3 |
| GCGR | 6WPW | G protein and NB35 | ZP3780 | 41.9 | 4 |
| GLP-1R | 5VAI | G protein and NB35 | GLP-1 | 50.9 | 5 |
| GLP-1R | 5NX2 | Truncated peptide | N-terminus Peptide 5 Agonist | 43.1 | 6 |

***^a^*** *Full-length construct*

***^b^*** *Distance measurements are performed from Cα to Cα for GCGR (L49 to C287) and GLP-1R (P56 to Y289).*

**Supporting Text**

**Functional assay of labeled GCGR and glucagon peptide**

We used steady-state FRET to explore the interaction between GCGR and Alexa Fluor 647 labeled glucagon (Figure S2A). A 100 nM donor labeled GCGR was used to measure the fluorescence emission in the absence and presence of acceptor labeled glucagon. We observed a donor-only emission peak (567 nm) for GCGR upon excitation at 550 nm (Figure S3*A*, red). In the presence of 1 µM Alexa Fluor 647 labeled glucagon, we found that the donor peak decreased and the appearance of an acceptor emission peak at 666 nm, indicating strong energy transfer from the donor to the acceptor (Figure S3*A*, green). We titrated acceptor-labeled glucagon from 1 nM to 10 μM and monitored the donor and acceptor emissions. The fluorescence emission spectra showed a gradual decrease in the donor fluorescence with a simultaneous increase of the acceptor fluorescence at 666 nm. This confirmed glucagon binding to GCGR. Relative FRET efficiencies were calculated as $E_{FRET}={I_{A}}/{(I_{D}+I_{A})}$, where *I*_D_ and *I*_A_ are the fluorescence intensities of the donor and the acceptor, respectively. The global dissociation constant (KD = 240.6 ± 16.1 nM) for glucagon was calculated by plotting FRET as a function of glucagon concentration and fitting to the Hill equation (Figure S3*B*, red). The observed KD value is comparable with previously reported IC50 values for similar full-length GCGR constructs (2,7). As a negative control, we used Cy5 labeled dermorphin, a peptide agonist for the µ-opioid receptor (Figure S2B) (8,9). As expected, we did not observe any appreciable increase in FRET values at any dermorphin concentrations (Figure S3*B*, green points). These results indicate a specific interaction between GCGR and glucagon, suggesting that the labeled GCGR is active and retains ligand binding activity when purified using detergent micelles.

**Supplementary References:**

1. Wu, F., Yang, L., Hang, K., Laursen, M., Wu, L., Han, G. W., Ren, Q., Roed, N. K., Lin, G., Hanson, M. A., Jiang, H., Wang, M. W., Reedtz-Runge, S., Song, G., and Stevens, R. C. (2020) Full-length human GLP-1 receptor structure without orthosteric ligands. *Nat Commun* **11**, 1272

2. Zhang, H., Qiao, A., Yang, D., Yang, L., Dai, A., de Graaf, C., Reedtz-Runge, S., Dharmarajan, V., Zhang, H., Han, G. W., Grant, T. D., Sierra, R. G., Weierstall, U., Nelson, G., Liu, W., Wu, Y., Ma, L., Cai, X., Lin, G., Wu, X., Geng, Z., Dong, Y., Song, G., Griffin, P. R., Lau, J., Cherezov, V., Yang, H., Hanson, M. A., Stevens, R. C., Zhao, Q., Jiang, H., Wang, M. W., and Wu, B. (2017) Structure of the full-length glucagon class B G-protein-coupled receptor. *Nature* **546**, 259-264

3. Zhang, H., Qiao, A., Yang, L., Van Eps, N., Frederiksen, K. S., Yang, D., Dai, A., Cai, X., Zhang, H., Yi, C., Cao, C., He, L., Yang, H., Lau, J., Ernst, O. P., Hanson, M. A., Stevens, R. C., Wang, M. W., Reedtz-Runge, S., Jiang, H., Zhao, Q., and Wu, B. (2018) Structure of the glucagon receptor in complex with a glucagon analogue. *Nature* **553**, 106-110

4. Hilger, D., Kumar, K. K., Hu, H., Pedersen, M. F., O'Brien, E. S., Giehm, L., Jennings, C., Eskici, G., Inoue, A., Lerch, M., Mathiesen, J. M., Skiniotis, G., and Kobilka, B. K. (2020) Structural insights into differences in G protein activation by family A and family B GPCRs. *Science* **369**

**5.** Zhang, Y., Sun, B., Feng, D., Hu, H., Chu, M., Qu, Q., Tarrasch, J.T., Li, S., Sun Kobilka, T., Kobilka, B.K., and Skiniotis, G. (2017) Cryo-EM structure of the activated GLP-1 receptor in complex with a G protein. *Nature* **546**, 248-253

**6.** Jazayeri, A., Rappas, M., Brown, A.J.H., Kean, J., Errey, J.C., Robertson, N.J., Fiez-Vandal, C., Andrews, S.P., Congreve, M., Bortolato, A., Mason, J.S., Baig, A.H., Teobald, I., Dore, A.S., Weir, M., Cooke, R.M., and Marshall, F.H. (2017) Crystal structure of the GLP-1 receptor bound to a peptide agonist. *Nature* **546,** 254-258

**7.** Siu, F. Y., He, M., de Graaf, C., Han, G. W., Yang, D., Zhang, Z., Zhou, C., Xu, Q., Wacker, D., Joseph, J. S., Liu, W., Lau, J., Cherezov, V., Katritch, V., Wang, M. W., and Stevens, R. C. (2013) Structure of the human glucagon class B G-protein-coupled receptor. Nature 499, 444-449

**8.** Kuszak, A. J., Pitchiaya, S., Anand, J. P., Mosberg, H. I., Walter, N. G., and Sunahara, R. K. (2009) Purification and functional reconstitution of monomeric mu-opioid receptors: allosteric modulation of agonist binding by Gi2. J Biol Chem 284, 26732-26741

**9.** Giakomidi, D., Bird, M. F., McDonald, J., Marzola, E., Guerrini, R., Chanoch, S., Sabu, N., Horley, B., Calo, G., and Lambert, D. G. (2021) Evaluation of [Cys(ATTO 488)8]Dermorphin-NH2 as a novel tool for the study of mu-opioid peptide receptors. PLoS One 16, e0250011
